# Comparative Analysis of the Thermal Conductivity of Handmade and Mechanical Bricks Used in the Cultural Heritage

**DOI:** 10.3390/ma15114001

**Published:** 2022-06-04

**Authors:** Alejandro Cabeza-Prieto, María Soledad Camino-Olea, María Paz Sáez-Pérez, Alfredo Llorente-Álvarez, Ana Belén Ramos Gavilán, María Ascensión Rodríguez-Esteban

**Affiliations:** 1 E.T.S. de Arquitectura, Universidad de Valladolid, avda Salamanca, 18, 47014 Valladolid, Spain; mcamino@arq.uva.es (M.S.C.-O.); llorente@arq.uva.es (A.L.-Á.); 2 Campus Fuentenueva, Departamento de Construcciones Arquitectónicas, Universidad de Granada, calle Severo Ochoa, s/n, 18071 Granada, Spain; 3 Campus Viriato, Universidad de Salamanca, Avda Cardenal Cisneros, 34, 49001 Zamora, Spain; aramos@usal.es (A.B.R.G.); mare@usal.es (M.A.R.-E.)

**Keywords:** thermal conductivity, heat flow tests, bricks, brick masonry, energy efficiency, cultural heritage

## Abstract

During interventions to improve the energy efficiency of cultural heritage, it is common to use methodologies that are used for current buildings with different thermal behaviour. For this reason, research has been carried out on the thermal behaviour of old brick walls by carrying out thermal flow tests in the laboratory on brickwork specimens, in order to compare the behaviour of handmade bricks and mechanical bricks from more than a century ago, and to analyse the relationship between the values of thermal conductivity, humidity, density and porosity, as well as to compare these results with those obtained by applying the procedure of the EN-1745 standard. It was concluded that bricks behave thermally differently, depending on the manufacturing process: handmade or mechanical, in both types of brick it was found that the higher the moisture content and density were, the higher the brick’s thermal conductivity value. It has also been concluded that old bricks have thermal conductivity values different from those indicated in EN-1745 as a function of density, and that the ratio detected in these specimens in the dry state and in the wet state does not conform to the processes indicated in the standard. With regard to porosity, it is important to note that the greater the closed porosity, the lower the conductivity. It has been concluded that in order to intervene in cultural heritage buildings, it is necessary to carry out a specific study of the behaviour of the systems with which they were constructed.

## 1. Introduction

The conservation of cultural heritage is of great interest to society for cultural, social and economic reasons, but at the present time it is necessary to find solutions to improve the energy efficiency of these buildings so that their conservation is also of interest from an environmental point of view. Sustainable management of cultural heritage is a strategic option contemplated by the European Union, including energy efficiency. For these reasons, in recent years, various committees and research teams developed guidelines to improve energy efficiency, such as EN 16883 [1] and publications [2,3,4,5]; even the European Union has funded projects such as HeLLO [6], Co2olBricks [7] or 3ENCULT [8], in which various proposals were developed in order to improve energy efficiency, especially in buildings constructed with brick. In general, they are based on parameters, such as the transmittance of the envelopes, and on improving this parameter with construction solutions that include the attachment or inclusion of thermal insulation sheets in the envelopes.

Although advances in the specific study of the behaviour of materials and construction systems of cultural heritage buildings are significant, standards and simulation programs often use the same methods and starting data as those for new buildings, when these buildings are constructed with very different materials and construction systems than the current ones; hence, their thermal performance is different [9]. Another factor to be studied is that the materials used in each geographical area tend to have peculiar characteristics, thus it is important to carry out local studies on the behaviours of these materials, in order to provide information for future interventions.

**Figure 1:**
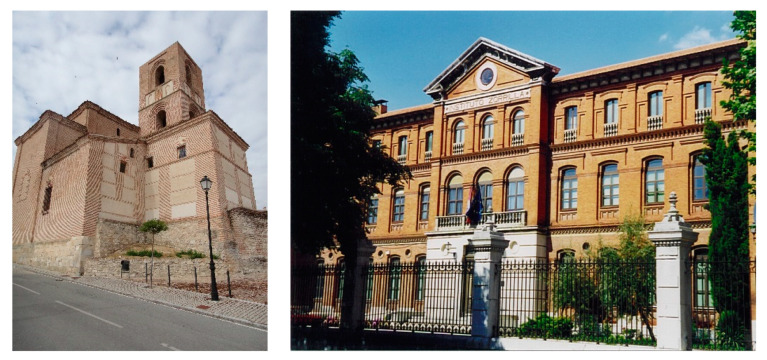
On the left, Mudejar church of Arévalo, Ávila (handmade bricks), and on the right, Institute Zorrilla, Valladolid (mechanical bricks).

In order to better understand the thermal behaviour of handmade brick cultural heritage buildings in a central area of Castilla y León, Spain, in the continental climate zone Csb and Csa, according to the Köppen climate classification [10], where the use of this material has been a constant throughout history [11,12] (see Figure 1), several investigations have been carried out in which a relationship has been established between the conductivity of these ancient masonries, and the brick density and moisture content; thus, this information can be used to better understand their thermal behaviour [13,14,15,16,17].

In the construction of cultural heritage buildings over the last two centuries, mechanical bricks have been used in preference to the handmade bricks characteristic of older buildings; they have different properties due to their mechanical manufacturing process. For this reason, a campaign of tests has been carried out with these types of bricks, using a similar methodology [13] to that carried out with handmade bricks, in order for the behaviour of handmade bricks and mechanical bricks to be comparatively analysed in terms of the relationship of thermal conductivity with brick density and moisture content of the masonries, and to check whether the results of the investigations with handmade bricks [9] can be extrapolated to mechanical bricks.

Previous research with handmade bricks [13] has analyzed the relationship between conductivity, density and water content. This relationship obviously influences the performance of a facade made of porous materials that can absorb significant amounts of rainwater [18,19,20]. In this research, real and apparent porosity is also included in the study, since this characteristic can be a determinant in thermal conductivity [21,22,23], and is different in handmade bricks and mechanical bricks. These characteristics of bricks are also analysed in this comparative study.

Numerous studies have been carried out on bricks of different types [24] in which the relationships between physical properties, such as porosity, absorption, etc., and their thermal behaviour, have been analysed. The volume, size and shape factor of the pores are determining factors in permeability and thermal behaviour, however, no research has been found on bricks manufactured in the study area that relate thermal conductivity to density and porosity; thus, it was considered appropriate to carry out this research.

The final objective of the tests is to compare samples of brick masonry made with three different types of brick previously characterized, and to know the relationship between conductivity, density and porosity of the brick, and the water they could contain; it remains to be seen if the relationships between these characteristics are similar for bricks made with different processes: by hand, by extrusion and pressing, and where the firing is also different: the first in Arab type kilns [25], and the others with industrial kilns [20]. In the same line, the comparison of results is established when the calculation of conductivity is determined by means of the test performed on samples, or on the basis of the applicable standard EN 1745 [26].

## 2. Materials and Methods

The research has been carried out in different phases, as detailed below.

### 2.1. Location and Selection of Study Material

The necessary material was obtained from buildings dating from the 19th and early 20th centuries, which at that time were being renovated or partially demolished, making it possible to obtain pieces from their walls. The sampling method from existing structures and the choice of bricks were based on the results of previous research carried out in the buildings, and the brick characteristics and manufacturing processes [11,27,28,29,30].

The materials obtained were selected according to their manufacturing process in 3 groups (handmade, extruded and pressed), in order to be able to relate their characteristics to the conductivity of the brick samples studied [30]. Within each group, the bricks were classified by type of brick into 6 subgroups, according to their origin and dimensions, with those of similar shapes being selected, which were subsequently used for the manufacture of the different test pieces. A total of 18 to 25 bricks were collected for each sample, with a total of 6 being made for this study, as described below.

**Figure 2:**
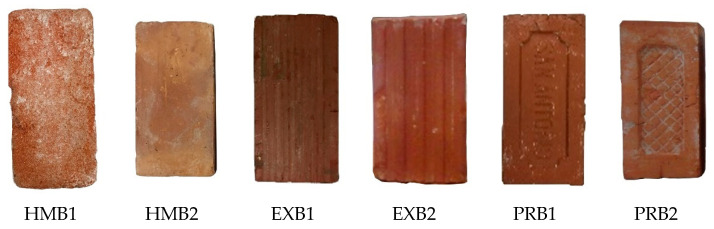
Photographs and denomination of the different bricks.

The designation of the pieces, according to the manufacturing process and type of brick, is shown in Figure 2: HMB (hand-made brick), EXB (extruded brick) and PRB (pressed brick).

### 2.2. Characterization of Brick Pieces

The characterization tests performed on the bricks were the following:

- Measurement of dimensions UNE-EN 772-16 [31].
- Dry bulk density according to UNE-EN 772-13 standard process [32].
- Porosity according to standard EN 772-3 [33].
- Mercury porosimetry ASTM D4404-18 [34,35].

The samples were prepared using CL90 air lime and washed river sand of controlled grain size, which was sieved with ASTM No. 4 mesh (<4.75 mm).

For their characterization, 12 samples of air lime and sand in 1/3 proportion were manufactured following the recommendations of old masonry treaties [36], with dimensions 40 × 40 × 160 mm. These samples were tested according to the same standards as those specified for the bricks, except for the density test, which was performed according to EN 1015-10 [37].

### 2.3. Manufacture of Test Samples

In this phase, six test samples were built from the selected bricks, and with the described mortar. On these samples, heat flow plate tests were performed according to ISO 9869-1 [38].

The use of whole brick pieces of different sizes conditioned the final dimensions of the samples and the thickness of the mortar joints, having to place between four and five courses in order to achieve a similar height between them, and a volume ratio between bricks/mortar of 70%/30%. The joints were adjusted to the measurements of the bricks, in order to achieve a similar proportion in all the samples.

The use of the same type of mortar avoided deviations derived from the change in the material of the joints. Likewise, the samples were made in such a way that the proportion of brick and mortar was similar in all of them, since the thermal conductivity of a masonry depends on the materials with which it is built, as indicated in standard EN 1745 [26].

Once the samples had been made, they were measured and weighed in dry state. The samples were then immersed in water, and once saturated, were reweighed to calculate the water absorbed following the standard used for brick EN 772-21 [39].

### 2.4. Heat Flow Tests

A refrigerated chamber, highly insulated with 10-centimeter-thick extruded polystyrene sheets (thermal conductivity λ = 0.033 (W/m–K)), was used for the thermal flow test. The refrigerated chamber was located in the same laboratory where the samples were made. In order to emulate the cold environment outside the buildings, a compact Zanotti MGM10328F cold production equipment was placed inside the chamber, with an evaporator inside, in order to maintain the environment at a controlled temperature, between 0 and 5 °C. The laboratory temperature during the test was between 20 °C and 22 °C, with humidity between 40% and 50%.

The heat flow test, which has been developed in similar studies [40,41,42], was carried out following the procedure of ISO 9869-1 [38]. The test was initiated once the water-saturated samples were placed on two opposite walls of the chamber provided for this purpose. During the test, the samples were allowed to dry, and weight measurements were taken at weekly intervals, so that data were available to relate the heat flow value and surface temperatures to their water content.

**Figure 3:**
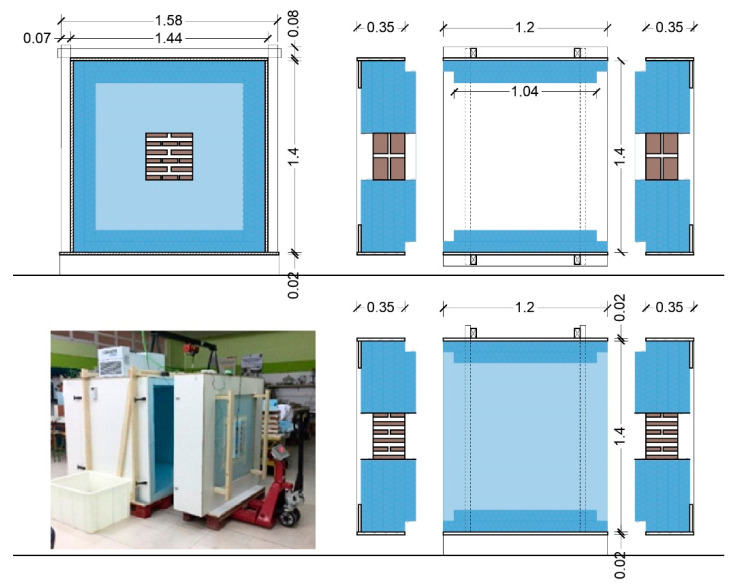
Above left, elevation of the cold chamber. Top right, floor plan. Bottom left, photograph. Bottom right, vertical section.

The brick masonry samples were in contact with the laboratory environment on one side, and with the refrigerated environment inside the chamber on the other side, achieving the effect of real thermal exposure of a facade wall. In order to avoid perimeter heat transmission to the samples, these two walls were built with a thickness greater than that of the samples, using four insulation panels, while the other two, the ceiling and the floor, were built with only two panels. Figure 3 shows a graphic diagram of the chamber. The chamber was designed to have two doors facing each other that could be displaced to place the samples. Figure 3 shows the displaced door to access the interior and the back of the sample.

For the test, a heat flow plate and two surface temperature probes were placed on the inside and outside of the sample. The trial lasted between three and six months, depending on the time required to complete the desorption process. The instrumental equipment used to carry out this heat flow test are listed in Table 1.

According to ISO 9869-1 [29], with the data obtained in the heat flow test (surface temperatures and the value of the heat flow through each sample), the value of the thermal conductance can be calculated using the formula of the simplified average method.

(1) $$\Lambda =\frac{\sum_{j=1}^{n}q_{j}}{\sum_{j=1}^{n}\left(T_{sij}-T_{sej}\right)}$$

where:

*Λ*: thermal conductance, W/(m^2^·K);

*Q*: density of heat flow rate = Φ/A, W/m^2^;

*T_si_*: inside surface temperature, °C;

*T_se_*: outside surface temperature, °C.

Once the value of the thermal conductance is known, the value of the thermal conductivity can be estimated using the formula:

*Λ* = *Λ* × d (2)

where:

*λ*: thermal conductivity (W/m·K);

*d*: thickness of the sample (m).

With the value of the thermal conductivity of each sample in each phase of the test, and the water content in m^3^/m^3^ obtained by weighing the samples, it was possible to establish the relationship between both values for each sample tested, and to analyse the relationship between the characteristics of the bricks, the water content and the value of the thermal conductivity of the sample.

### 2.5. Analytical Calculation According to Standard UNE-EN 1745

In performing the analytical calculation, the first step was to determine the behaviour of the samples, making an estimation based on the procedure of EN 1745 [26]. In a second step, it was checked whether the two procedures followed (in the laboratory by means of flow tests and by analytical calculation according to the standard) obtained similar results for the old masonries [43].

The analytical calculation process, as established by EN 1745 [26], indicates that the design thermal conductivity values of a constructed masonry is a function of the values of the component elements; in this case, the design thermal conductivity of brick and mortar percentage, and the area in elevation of both materials. The following formula was applied:

(3) $$\;\lambda_{desing,\;mas}=a_{unit}\;\times \;\lambda_{design,unit}+a_{mor}\;\times \;\lambda_{design,mor}\;$$

where:

*λ_design,mas_*: masonry design thermal conductivity;

*λ_design,unit_*: design thermal conductivity of brick;

a*_unit_*: percentage of the area in the elevation;

*λ_design,mor_*: design thermal conductivity of mortar;

a*_mor_*: percentage of the area in the elevation.

In the case of the test samples under study, the ratio of brick/average joint was considered to be 70/30%; with this value, estimates were made from the value of the thermal conductivity of the material in the dry state, and at 10° C average temperature.

The corresponding design thermal conductivity value was then calculated using the moisture conversion factor, which is calculated using the moisture conversion coefficient and the moisture content.

(4) $$\;\lambda_{desing}=\lambda_{10,dry}\times e{\;}^{f_{\psi }\;\times \;\psi_{desing}\;}$$

where:

*λ_design_*: masonry design thermal conductivity;

*λ*_10,*dry*_: thermal conductivity of the material in the dry state and at 10 °C average temperature;

*e*: moisture conversion factor;

*f_ψ_*: moisture conversion coefficient;

*ψ_design_*: moisture design content.

In order to carry out the estimation, the thermal conductivity values of the Table A1 of the standard EN 1745 [26] were taken from solid pieces of fired clay for different densities: for ρ = 1700 kg/m^3^, *λ*_10,*dry,mat*_ = 0.51 W/(m·K); ρ = 1800 kg/m^3^, *λ*_10,*dry,mat*_ = 0.55 W/(m·K); ρ = 1900 kg/m^3^, *λ*_10,*dry,mat*_ = 0.60 W/(m·K); ρ = 2000 kg/m^3^, *λ*_10,*dry,mat*_ = 0.64 W/(m·K); and for the mortar *λ*_10,*dry,mat*_ = 0.79 W/(m·K). Finally, the humidity conversion coefficient by volume is *f_ψ_* = 10 for the brick and *f_ψ_* = 4 for the mortar if the water content of the samples is given in m^3^/m^3^.

In order to perform this last calculation, it was necessary to determine the temperature conversion coefficient, since the dry density calculation temperature according to Table A1 of the standard EN 1745 [26] established at 10 °C did not coincide with the average laboratory temperature, which was 22 °C.

EN ISO 10456 [44] gives the relation of the thermal conductivity at different temperatures as a function of a temperature conversion coefficient, which for fired clay and mortar corresponds to the following expression:

(5) $$F_{T}={e\;}^{f_{T}\;\left(T2-T1\right)}$$

where:

*F_T_*: thermal conductivity;

$e^{f_{T}}$: temperature conversion factor;

*T*: temperature.

For the materials used and the temperature difference, *F_T_* = 1.01.

Finally, the thermal conductivity (calculation) was obtained from the linear extrapolation of the thermal conductivity values established by the standard for the density values of the samples adapted to the theoretical test conditions, according to the methodology described above.

### 2.6. Data Analysis and Comparative Study

In the last phase, the results of the two systems were analysed and compared [43]. Those obtained from the flow test and those resulting from applying the data and formulation of the EN 1745 standard [26] were used, in order to establish the relationship between the thermal behaviour of the masonry and the characteristics of the bricks with which they were built. This type of information will allow to know very important data before an intervention in cultural heritage. It will be possible to have information about the materials with which it was built, and not depend on information based on modern materials.

## 3. Results

The results are presented in the same order in which the tests were carried out to obtain them: first, the results for the bricks and mortar; then, the results of the thermal flow tests on the samples; finally, the analytical results according to EN 1745 [26], and then a comparison of the test results with those obtained analytically.

### 3.1. Results of Tests Performed on Bricks

**Figure 4:**
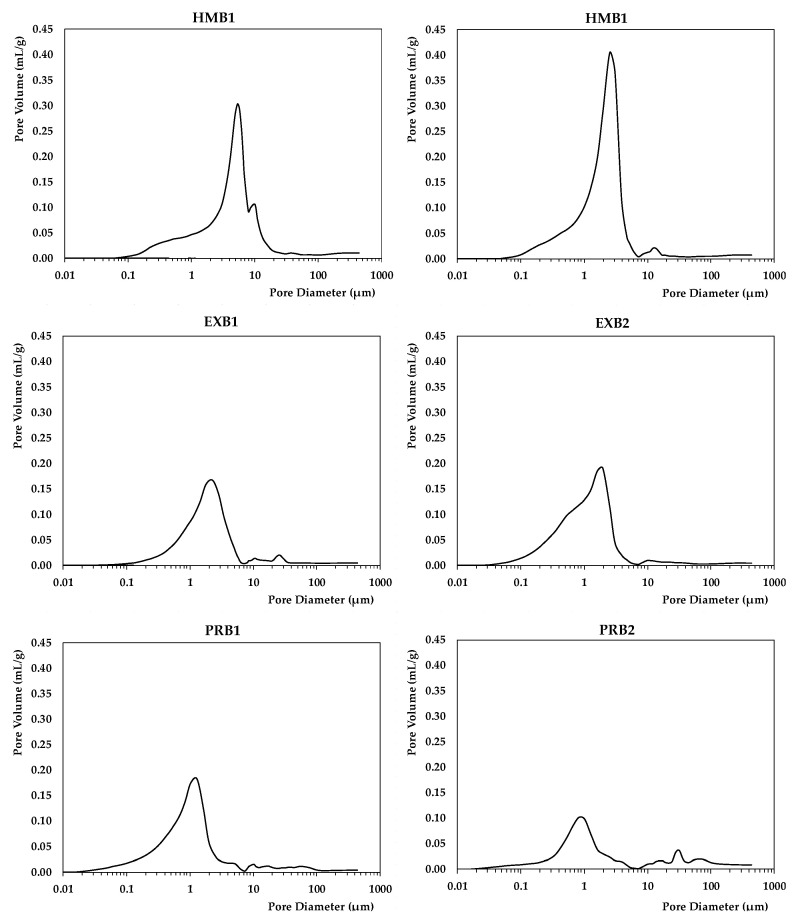
Pore size distribution in samples corresponding to the three types of brick studied.

Table 2 shows the results of the physical tests related to the dimensions of the parts and their density. Table 3 and Figure 4 show the results of the mercury porosimetry test.

The values in Table 2 and Table 3 are the results of tests on the different types of bricks used to construct the sample, which, as indicated above, have the most similar characteristics among the bricks recovered.

Figure 4 below shows the mercury injection porosimetry test plots for some of the tested brick samples.

### 3.2. Results of Tests on Test Samples

Table 4 shows the results of the physical tests carried out on the samples: dimensions, bulk density, water absorption, water content and water absorption.

**Figure 5:**
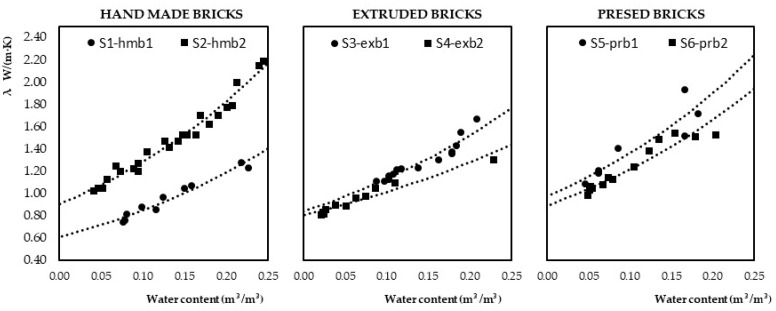
Results of heat flow and water content tests of brick masonry samples.

The results of the thermal flow tests performed on the samples are shown in Figure 5. With this, as shown in Table 5, it was possible to determine the conductivity value as a function of the water content of each sample.

From the formulas of the calculated trend lines and known values of dry conductivity (for a water content of 0) and wet conductivity (for a maximum water content of the samples), the relationship between the apparent porosity and the real porosity of the bricks of the tested samples was determined.

### 3.3. Results of the Analytical Study

This section presents the results of the analytical study carried out based on the application of standard EN 1745 [20]. Table 6 shows the dry and saturated conductivity values of the test samples resulting from the calculation process, according to the methodology described above.

**Figure 6:**
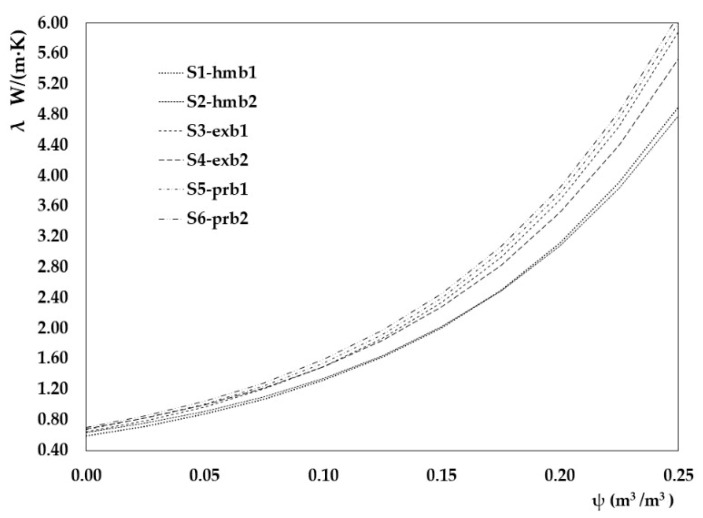
Results of the analytical calculation of the conductivity of dry samples with different moisture contents according to the different densities established in the EN 1745 standard [26].

In order to facilitate the understanding of the study, Figure 6 shows a graph similar to those included in Figure 5, which establishes the relationship between conductivity, density and water content of the test samples, according to the result obtained from the calculation carried out according to the standard, according to the density of the brick in Table 2 and the brick/mortar ratio Table 4.

Finally, Table 7 shows the correlation formulas for thermal conductivity as a function of water content to each type of bricks. They have been included with the trend lines test results and those of the analytical calculation (corresponding with Figure 5 and Figure 6, respectively).

## 4. Discussion

The results of the flow tests used to calculate the conductivity as a function of humidity, Figure 5, show the following, from the three groups of test samples:

1. The higher the water content, the higher the conductivity.
2. For the same water content, the higher the density value, the higher the conductivity.
3. Quantitatively, the conductivity results obtained for each group of test samples are different, which determines that the manufacturing process of the bricks used influences the behaviour of the set.

When each group of samples was analysed independently (Table 8), the following results were found:

1. The samples with handmade bricks with lower density show a reduction of up to 40% of the conductivity value for the same water content as the denser ones.
2. In the samples with extruded and pressed bricks, these differences are less than 10% in the first case and 1% in the second.

**Table 8:** Comparison of conductivity values in dry and saturated states for the samples studied by the applied methods.

Samples		S1-hmb1	S2-hmb2	S3-exb1	S4-exb2	S5-prb1	S6-prb2
Apparent density (kg/m^3^)		1692	1730	1830	1827	1911	1894
Water abpsortion (m^3^/m^3^)		0.242	0.245	0.253	0.237	0.223	0.184
*λ_dry_* W(m·K)	Calculation	0.59	0.61	0.65	0.67	0.68	0.71
*λ_dry_* W(m·K)	Test	0.61	0.91	0.84	0.93	0.89	0.98
*λ_satured_* W(m·K)	Calculation	4.61	4.93	5.83	5.26	4.64	3.45
*λ_satured_* W(m·K)	Test	1.35	2.14	1.79	1.94	1.78	1.79

For a more detailed analysis, the pore size distribution of the samples was examined (Figure 4), and showed that different pore sizes are observed for each group. In the case of the handmade brick, the proportion of pores (<1 µm) is much smaller than for the other two types of brick, with the mean diameter being 2.11 µm. Extruded bricks have a mean diameter of 0.80 µm, with a 0.54 µm mean diameter for pressed bricks.

The observed behaviour seems to indicate that non-accessible porosity, i.e., pores that do not communicate with each other, has an important influence on the conductivity values, and that density is not the only decisive factor in the behaviour of the masonry, especially when it has a water content >0%.

Finally, comparing the conductivity results obtained with the flow test and applying the standard procedure, it can be seen that the results are different in all cases. In the case of the conductivity for the tile brick sample S1-hmb1, the maximum difference is reached, since the value of the conductivity in this sample’s saturated state, according to the calculation performed, is approximately 3.38 times higher than the value of its conductivity resulting from the conductivity in the laboratory test. For the extruded brick sample S3-exb1, it is 3.38 times higher, while for the pressed brick sample S5-pr1b, it is 2.60 times higher.

The main cause, for these differences, is recognized in the manufacturing system. Undoubtedly, this generates different characteristics between the old and current brick types due to their elaborations.

A second cause is a direct consequence of the calculation procedure established in the standard, which assigns a moisture conversion factor for fired clay pieces of 10, without exception, and 4 for mortar. The result is that the value of the exponent that multiplies the water content ranges from 3 to 3.5 in the results of the test, and from 8.51 to 8.88 in the calculation. This aspect will require further testing in which the different types of bricks’ behaviour will have to be analysed.

## 5. Conclusions

The article focuses on the comparative study of the thermal conductivity of different types of bricks used in cultural heritage. At the same time, the physical properties that characterize the material through the tests carried out are compared, which allows for the establishment of necessary specifications for the repair and replacement of bricks in the facades that could be used in the restoration process.

As a result of the study, the following conclusions can be obtained:

- The thermal conductivity of brick masonry presents higher values with higher moisture content, although the results of the tests carried out do not give values as high as those when the calculation is made according to the EN 1745 standard. Possibly, the moisture conversion factor given in the standard is very high for old bricks recovered in building interventions.
- The conductivity value also depends on the density and porosity. If bricks made or manufactured by the same process are compared, the higher the density, the higher the conductivity value. However, the same statement cannot be made when analysing handmade bricks with mechanical bricks (extruded and pressed).
- Another characteristic that influences conductivity is porosity. In the tests carried out, it was observed that there is a significant difference in the ratio between the real porosity and the apparent porosity between handmade bricks and mechanical bricks. In the handmade bricks, there is not as much difference between the two porosities as there is in the mechanical bricks, and it seems that this value may indicate the difference between the conductivity graphs to the water content of the samples made with different bricks. In the dry state, the difference in conductivity of mechanical brick samples is smaller in proportion to their density than that of handmade bricks. In the dry state, the difference in conductivity of mechanical brick samples is smaller in proportion to their density than that of handmade bricks.
- When analyzing the conductivity values in dry and saturated states, the handmade brick with the lowest density performed better in the test, with lower thermal conductivity values in the dry state.
- If the results of the laboratory tests are compared with those that have been estimated by the EN 1745 procedure based on the densities of the bricks, it can be concluded that, in the dry state, the conductivity values, resulting from the tests, are higher; however, this values, in the saturated state, are much lower. This could indicate that the values used according to the current standards are not appropriate for the historical brick masonry.
- These results obtained are important for the energetic rehabilitation of old buildings built with bricks, since they allow us to know the real values of conductivity of this type of masonry, and evaluate the importance of controlling the water content in the interventions, not only because of injuries that can occur, but also because the difference in conductance between dry and saturated walls can be from 1.82 to 2.21 depending on the type of brick; these differences can cause important errors in the study of the thermal behaviour of these buildings if this factor is not taken into account.

## Figures and Tables

**Table 1:** Characteristics of HFM apparatus used.

Instruments		
Heat flux plate	name	AMR model FQAD18TSI de Ahlborn
	shape of place	square
	dimensions	120 × 120 mm
	thickness	3 mm
	type of the substrate	Silicone
	accuracy of the measurement	±0.02%
	sensitivity of the instruments	Not specify
Surface temperature probes	Number of probes	2 surface temperature, inside and outside
	typology	thermocouple
	position	internal and external of camera
	range of the measurement	−00 a + 95 °C
	accuracy of the measurement	±0.05%

Source: elaboration of the authors from the manufactured manuals, Almemo.

**Table 2:** Material properties: dimensions, water absorption and apparent density.

Brick/Mortar	Dimensions (mm)						Apparent Density (kg/m^3^)	
	Length		Width		Thickness			
	Media	Dev.	Media	Dev.	Media	Dev.	Media	Dev.
HMB1	302	3	148	3	40	3	1676	16
HMB2	250	5	122	4	52	3	1735	55
EXB1	234	3	123	1	56	1	1865	23
EXB2	257	4	137	2	48	2	1922	63
PRB1	261	1	127	1	53	1	1934	17
PRB2	224	3	109	2	53	1	2044	95
mortar	160	2	40	1	40	1	1729	20

**Table 3:** Material properties: apparent porosity, brick porosity and average pore size.

Brick/mortar	Apparent porosity (%)	Total Porosity (%)	Ø pore media (μm)
HMB1	25.17	26.94	1.59
HMB2	25.37	27.51	2.63
EXB1	25.72	29.18	1.01
EXB2	22.49	29.50	0.59
PRB1	22.02	26.19	0.44
PRB2	16.68	20.91	0.64
mortar	23.06	28.04	1.04

**Table 4:** Test results of test samples.

Sample	S1-hmb1	S2-hmb2	S3-exb1	S4-exb2	S5-prb1	S6-prb2
Photographs	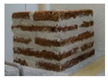	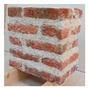	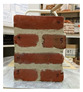	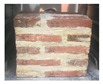	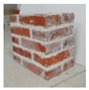	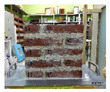
Dimensions (cm^3^)	30 × 30.5 × 25.5	38.5 × 25.5 × 39	25 × 27 × 25	41 × 34.5 × 28.5	41 × 31 × 26	34 × 32 × 23.5
Proportion by volume Brick/mortar	69/31%	61/39%	74/26%	63/37%	71/29%	68/32%
Apparent density (kg/m^3^)	1692	1730	1830	1827	1911	1894
Water absorption (m^3^/m^3^)	0.242	0.245	0.253	0.237	0.223	0.184

**Table 5:** Dry and saturated conductivity of the samples, test results, and ratio between the apparent and real porosity of the bricks used to make the samples.

Sample	S1-hmb1	S2-hmb2	S3-exb1	S4-exb2	S5-prb1	S6-prb2
*λ_dry_* W(m·K)	0.61	0.91	0.84	0.93	0.89	0.98
*λ_satured_* W(m·K)	1.35	2.14	1.79	1.94	1.78	1.79
Apparent porosity/Total porosity %	93.43	92.22	86.28	76.24	84.04	79.77

**Table 6:** Dry and saturated conductivity of the test samples, estimated according to EN 1745 [26].

Sample	S1-hmb1	S2-hmb2	S3-exb1	S4-exb2	S5-prb1	S6-prb2
*λ_dry_* W(m·K)	0.58	0.61	0.63	0.75	0.66	0.68
*λ_satured_* W(m·K)	4.56	4.58	6.05	4.91	4.64	3.33

**Table 7:** Correlation formulas for thermal conductivity as a function of water content in the different types of bricks.

Sample	S1-hmb1	S2-hmb2	S3-exb1	S4-exb2	S5-prb1	S6-prb2
*λ* W(m·K) test	$0.61\;e^{3.3\psi }$	$0.91\;e^{3.5\psi }$	$0.84\;e^{3.0\psi }$	$0.93\;e^{3.3\psi }$	$0.89\;e^{3.1\psi }$	$0.98\;e^{3.3\psi }$
*λ* W(m·K) calculation	$0.58\;e^{8.46\psi }$	$0.61\;e^{8.10\psi }$	$0.63\;e^{8.86\psi }$	$0.65\;e^{8.77\psi }$	$0.66\;e^{8.38\psi }$	$0.68\;e^{8.68\psi }$
